# Goals and organisational structure of the movement for global mental health

**DOI:** 10.1186/1752-4458-8-31

**Published:** 2014-07-23

**Authors:** Harry Minas, Alexandra Wright, Ritsuko Kakuma

**Affiliations:** 1 Global and Cultural Mental Health Unit, Centre for Mental Health, Melbourne School of Population and Global Health, The University of Melbourne, Melbourne, Australia; 2 Movement for Global Mental Health, Melbourne, Australia

## Abstract

**Background:**

The Movement for Global Mental Health (MGMH), established in 2008, is in a period of transition, as is the field of global mental health. The transfer of Secretariat functions from the Centre for International Mental Health to the Public Health Foundation of India was a suitable time to reflect on the goals of MGMH and on the form of organisational structure that would best serve the organisation in its efforts to achieve its goals.

**Methods:**

An online survey was sent to the 4,000 registered members of MGMH seeking the views of the membership on both the goals of MGMH and on the preferred form of organisational structure.

**Results:**

There was near unanimous (95%) agreement with the MGMH goals as stated at the time of the survey. The current form of organisation of MGMH, a loose network of individuals and organisations registered through the MGMH website, was the least preferred (29.9%) form of organisation for the future of MGMH. More than two thirds (70.1%) of respondents would prefer a formal legal structure, with 60% of this group favouring a Charitable Organisation structure and 40% preferring an international Association structure.

**Discussion:**

The response rate (7%) was too small and too skewed (predominantly academics and health professionals from high income countries) to allow any clear conclusions to be drawn from the survey. However, both the fact that responses were too few and skewed and the preferences expressed by respondents raise issues for careful consideration by the current MGMH Secretariat.

**Conclusions:**

The global mental health field and MGMH are in a time of transition. The move to the new secretariat is an opportunity for systematic consideration of the organisational structure and governance arrangements that will best serve the goals of MGMH.

## Background

The Movement for Global Mental Health (MGMH) [1-5] was established in 2008, following the publication of the first Lancet series on global mental health [6]. The goal of the Movement is to improve the availability, accessibility and quality of services for people with mental disorders by scaling up services based on scientific evidence and human rights. An informal secretariat was based in the NGO Sangath [7], and the work of establishing and managing MGMH was carried out entirely on a volunteer basis. The MGMH Advisory Group – the membership of which overlapped considerably with the Lancet Mental Health Group – made decisions about MGMH on a consensus basis.

In 2010 the Advisory Group decided that a secretariat was required to coordinate the activities of MGMH and called for applications from the MGMH membership. The Centre for International Mental Health (CIMH) [8], University of Melbourne (now the Global and Cultural Mental Health Unit, Centre for Mental Health), was selected as the MGMH Secretariat for three years (2011–2013). The responsibilities of the Secretariat included the management and daily administration of the website and social media platforms, membership applications, monthly newsletters, email queries to MGMH, organisation of the biennial Global Mental Health Summit and monitoring and evaluating the functions of MGMH.

The MGMH Secretariat continued to recruit members and carry out activities on a voluntary basis. In 2013 MGMH had a membership – individuals and organisations registered through the MGMH website - from over 60 countries, composed of 4,000 individuals and more than 100 organisations. Membership remains free and open to all, and includes health professionals, academics, students, government officers, NGOs, disabled people’s organisations, and people with mental disorders and their families or carers [9].

### MGMH online platforms

MGMH communicates with current and potential members in three main ways, via the MGMH website, monthly newsletters, and social media.

The MGMH website (http://www.globalmentalhealth.org) serves as the hub for communication and information about MGMH activities and news. Managed by the Secretariat, the website permits free membership registration and hosts the network of members and organisations that constitute the Movement. Importantly, the website has been designed to be open-source, which permits content to be created by registered users as well as by the Secretariat. This encourages members to post and discuss global mental health-related news, resources, publications and events.

Monthly MGMH newsletters are created from website content and recent global mental health news. Newsletters feature major announcements and events, job opportunities, recent publications, training opportunities, and media items related to global mental health.

MGMH maintains a Twitter account (@MGMentalHealth). With over 1,000 followers MGMH is able to quickly and regularly communicate with those interested in the Movement. These communications include announcements of relevant media items, newsletter releases, and new publications.

### The global mental health summit

MGMH holds a biennial Summit to bring together policymakers, academics, health professionals, consumers, carers, service providers, civil society organisations and disabled peoples’ organisations, particularly from low- and middle-income countries. These Summits provide opportunities to share experiences and expertise amongst the participants, promote MGMH and its objectives, foster new international linkages and collaborations, and to examine challenges and progress in global mental health development. The Summits have consistently included presentations and participation from representatives from a range of different countries and stakeholder groups (e.g., consumers, funders, policymakers).

The first Global Mental Health Summit was held in Athens in 2009, the second in Cape Town in 2011. The Cape Town Summit also saw the launch of the second Lancet series on Global Mental Health [10].

The third and most recent Global Mental Health Summit was held in Bangkok, Thailand in late August 2013. Held in association with the World Congress of Asian Psychiatry (WCAP), this Summit provided the opportunity for MGMH and participants to raise the visibility of important work in Asia to the global community and of the work of MGMH to the WCAP community. Session topics included the post-MDG development agenda, intersectoral partnerships, human rights, and the future organisation of MGMH. The session on the future of MGMH was informed by the results of the survey reported here. During the 3^rd^ Global Mental Health Summit Twitter was used to disseminate key points from Summit presentations to those who could not attend. Videos of the Summit presentations were also made available after the Summit [11].

### MGMH position statement on mental health in the post-2015 development agenda: Mental health is essential to achieve sustainable development

The upcoming deadline of the Millennium Development Goals has catalysed a global discussion about the post-2015 development agenda. A session at the 3^rd^ Global Mental Health Summit provided the platform to discuss the importance of a unified message by the global mental health community on this issue and highlighted the importance of advocating for the inclusion of mental health in future development programs. Discussion of a draft MGMH position statement on the post-2015 development agenda [12] was one of the key sessions in the Bangkok Summit.

### The future of MGMH

MGMH is an unfunded initiative with no formalised organisational status. It continues to rely completely on the commitment of its members to volunteer their time and resources to implement its activities, such as participating in the Summit Planning Working Group, developing the monthly MGMH newsletter, and submitting grant proposals to provide funding for members from low- and middle-income countries to attend the Global Mental Health Summits [9].

The term of the first Secretariat came to an end in December 2013 and, after a public call for expressions of interest in hosting the second Secretariat, the Secretariat functions were handed over in February 2014 to the Public Health Foundation of India. In preparation for this transition the Secretariat designed and conducted a survey of MGMH members in July 2013 with the aim of generating information that would serve to guide the new Secretariat in making decisions about the organisational structure that will most effectively support the effective pursuit of the goals of MGMH.

## Methods

The 4,000 MGMH members were invited to participate in an online survey that was delivered through SurveyMonkey [13] in July 2013. Respondents were asked to provide demographic and professional background information, including their primary affiliation or self-identification (service user, carer, civil society organization, non-government organization, health professional, service provider, government officer, academic, student or other).

Respondents were then asked whether they agreed (“yes” or “no” response) with the core goals of MGMH, stated as follows: “The Movement for Global Mental Health aims to improve the availability, accessibility and quality of services for people with mental disorders worldwide – especially in low- and middle-income countries – by scaling up services based on scientific evidence and human rights.” [5].

To determine opinions and preferences concerning the organisational structure of MGMH respondents were presented with three organisational options:

1) No change: MGMH continues as it has been in a loose structure that centers around a public website.

2) Establish MGMH as an Association: MGMH is established as a legally constituted international association.

3) Establish MGMH as a Charitable Organisation: MGMH is established as a legally constituted charitable organisation, registered as having a charitable purpose.

Each of the three options was followed by further information about its anticipated benefits and challenges, and steps that would be required to transform the MGMH structure (see Table 1), to ensure appropriate understanding of the different options and the implications of selecting each. Respondents were then asked 1) whether each option is “suited to the objectives of MGMH” (yes/no response) and 2) to rank the three options in order of preference (from 1–3) for the future organisational structure of MGMH.

**Table 1:** Three options for the organisational structure of the movement for global mental health

	**Anticipated benefits**	**Anticipated challenges:**	**Steps required:**
**OPTION 1: No Change**			
Description: MGMH continues as it has been in a loose structure that primarily centers around a public website.	• Little change is required	• MGMH cannot collect any funding for its activities	• Develop Terms of Reference outlining roles and responsibilities of the MGMH Secretariat and Advisory Group
		• The momentum of MGMH remains low	• Develop process for selecting the next Secretariat, and make decision
		• Understanding of the purpose and activities of MGMH remains limited	• Get consensus regarding the existence of an Advisory Group: If existence is supported, attain consensus regarding size, structure and responsibilities of the Advisory Group
		• Formal partnerships, endorsements, etc. with MGMH are not possible	
**OPTION 2: Establish MGMH as an Association**			
Description: MGMH is established as an independent international association	• MGMH would have full autonomy	• Establishing MGMH as an association would require significant human resources and funding, and is a lengthy process	• Choose an appropriate country in which to establish the association
Example: World Psychiatric Association http://www.wpanet.org	• MGMH could collect membership fees and other funds	• MGMH is no longer a 'social movement' or a grass-roots initiative	• Secure funding to register MGMH as an association
	• Greater accountability for MGMH activities, hence greater transparency to its members		• Development of an MGMH constitution which outlines its rules for operation, governance and formal membership
			• Register as an association in a chosen country
			• Develop a process for collecting membership fees, and procedures for when these fees can be waived
			• Develop rules of membership
			• Get consensus regarding size, structure and responsibilities of the Advisory Group
			• Develop process for selecting the next Secretariat, and make decision
**OPTION 3: Establish MGMH as a Charitable Organisation**			
Description: MGMH is established as a charitable organisation, which therefore is registered as having a charitable purpose	• MGMH can collect donations and other funds for future activities	• Establishing MGMH as a charitable organisation would require significant human resources and funding, and is a lengthy process	• Choose an appropriate country in which to establish the association
Example: Canadian Coalition for Global Health Research (CCGHR) http://www.ccghr.ca	• Greater accountability for MGM activities, hence greater transparency to its members	• MGMH is no longer a 'social movement' or a grass­roots initiative	• Secure funding to register MGMH as an association
			• Development of an MGMH constitution which outlines its rules for operation, governance and formal membership
			• Register as an association in a chosen country
			• Develop a process for collecting membership fees, and procedures for when these fees can be waived
			• Develop rules of membership
			• Get consensus regarding size, structure and responsibilities of the Advisory Group
			• Develop process for selecting the next Secretariat, and make decision

Descriptive analyses were carried out for demographic data, level of agreement with the aims of MGMH, opinions on the suitability of three options for MGMH organisational structure, and ranking of the three options. Means and standard deviations were calculated for continuous variables and counts and percentages for categorical variables. Significance of differences across primary affiliation groups and across country World Bank categories was tested by one-way ANOVA for continuous variables and chi-square test for categorical variables.

## Results

### Respondents

**Figure 1:**
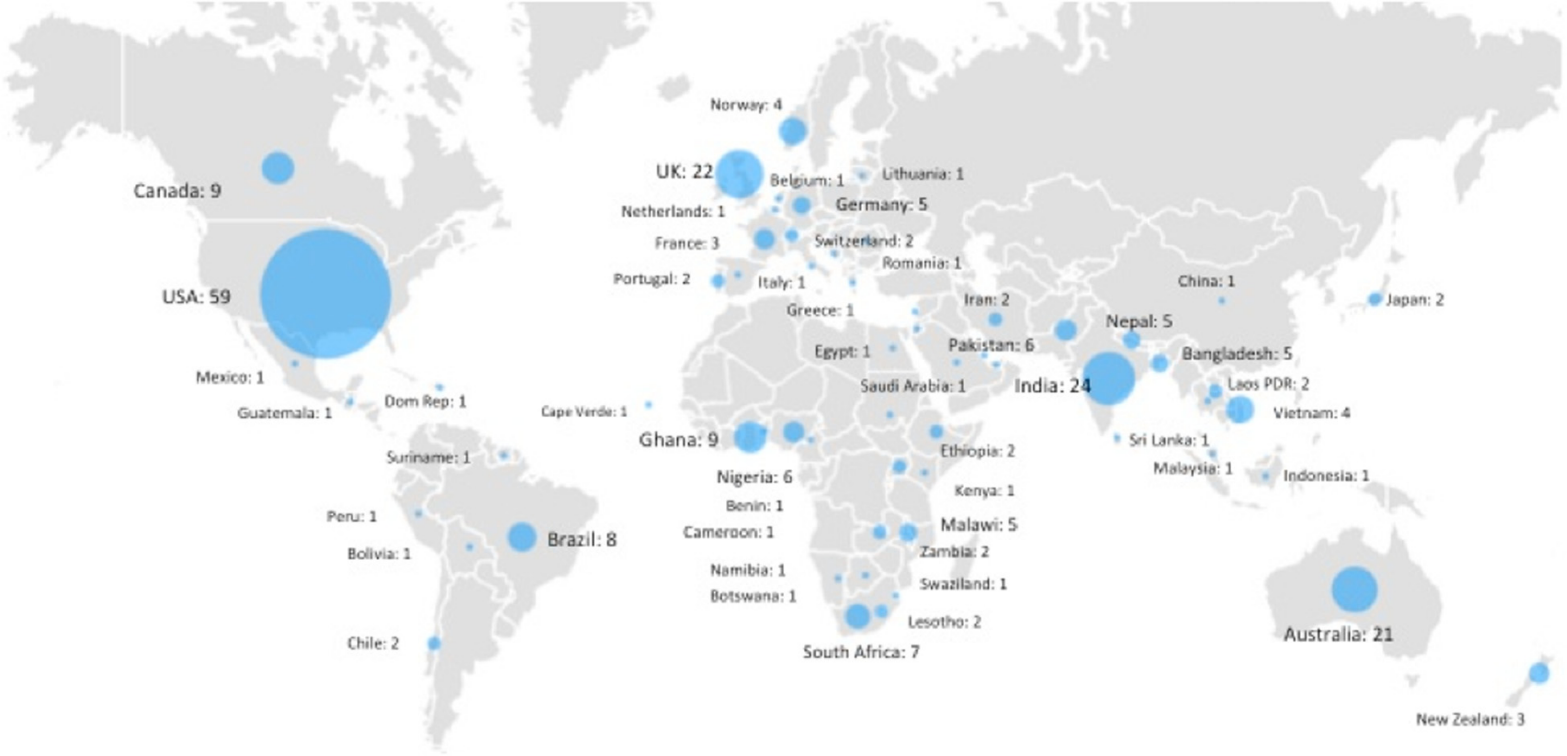
Countries of respondents.

From the 4,000 emails sent 279 responses were received, a response rate of 7.0%. Of the 279 respondents 40 respondents either did not give consent to publication or did not answer all survey questions, and were excluded from further analysis, leaving 239 responses with complete data.Respondents were from 61 countries, 29.0% of the world’s 210 countries - from 19 World Bank Category A (WB-A: high-income) countries, nine Category B (WB-B: upper-middle-income) countries, 17 Category C (WB-C: lower-middle-income) countries and 15 Category D (WB-D: low-income) countries. The geographic spread and number of respondents from each country are shown in Figure 1. The majority of respondents came from Category A (54.8%) and D (29.3%) countries, with very small numbers from Category B (8.8%) and C (7.1%) countries. There were 10 or more respondents from only four countries – USA (n = 59), India (n = 24) UK (n = 22) and Australia (n = 21).

There was approximately equal representation of males (51.4%) and females (48.6%) (Table 2). However, there was a significant (p < 0.01) difference in gender composition of respondents between World Bank Category A & B countries and World Bank Category C & D countries. The majority (58.2%) of WB-A & WB-B country respondents were female while the majority (68.1%) of WB-C & WB-D country respondents were male.

**Table 2:** MGMH survey data - descriptive data

	**Total sample**
**Demographics**	
Age (mean, SD)	44.78 (12.596)
Gender (N,%)	
Female	115 (48.1)
Male	124 (51.9)
Total	239 (100.0)
World Bank Category (N,%)	
1	131 (54.8)
2	21 (8.8)
3	17 (7.1)
4	70 (29.3)
Total	239 (100.0)
**Primary Role or Affiliation (N,%)**	
Service User or Carer	11 (4.6)
Community Organisation	42 (17.6)
Service Provider	76 (31.8)
Academic	84 (35.1)
Student or Other	26 (10.9)
Total	239 (100.0)
**Agreement with MGMH Goals (N,%)**	
Yes	227 (95.0)
No	12 (5.0)
Total	239 (100.0)
**Suitability of MGMH organizational options (N,%)**	
No Change	
Yes	115 (52.0)
No	106 (48.0)
Total	221 (100.0)
Association	
Yes	139 (62.9)
No	82 (37.1)
Total	221 (100.0)
Charitable Organisation	
Yes	144 (65.2)
No	77 (34.8)
Total	221 (100.0)
**Choice of each organisational option as the most preferred (N,%)**	
No Change	66 (29.9%)
Association	66 (29.9%)
Charitable Organisation	89 (42.2%)

The mean age of the total sample was 44.8 years, with the youngest respondent aged 22 years and the oldest aged 83 years (20–29 years, 10%; 30–39 years, 32%; 40–49 years, 23%; 50–59, 20%; 60+ years, 15%). The largest sub-group (32%) was the 30–39 year age group. There was no significant difference in mean age across the primary affiliation groups or across the World Bank category groups.

The largest primary affiliation groups were academics (n = 84) and health professionals (n = 55). Service users, carers, NGOs and civil society organisations, service providers, government officers, students and others were all represented but the numbers were small. The primary affiliation categories were aggregated into the following composite affiliation groups for analysis: Service User or Carer (n = 11); Community organisations (n = 42); Service Provider (n = 76); Academic (n = 84); Student/Other (n = 26) (Table 2).

### MGMH goals

95% of respondents agreed with the stated goals of MGMH, that “*The Movement aims to improve the availability, accessibility and quality of services for people with mental disorders worldwide – especially in low and middle-income countries – by scaling up services based in scientific evidence and human rights.”*[5] (Table 2) There was no significant difference across primary affiliation groups or across World Bank country category groups in the proportion of respondents who agreed with MGMH goals.

### Suitability of organisational options for the goals of MGMH

**Figure 2:**
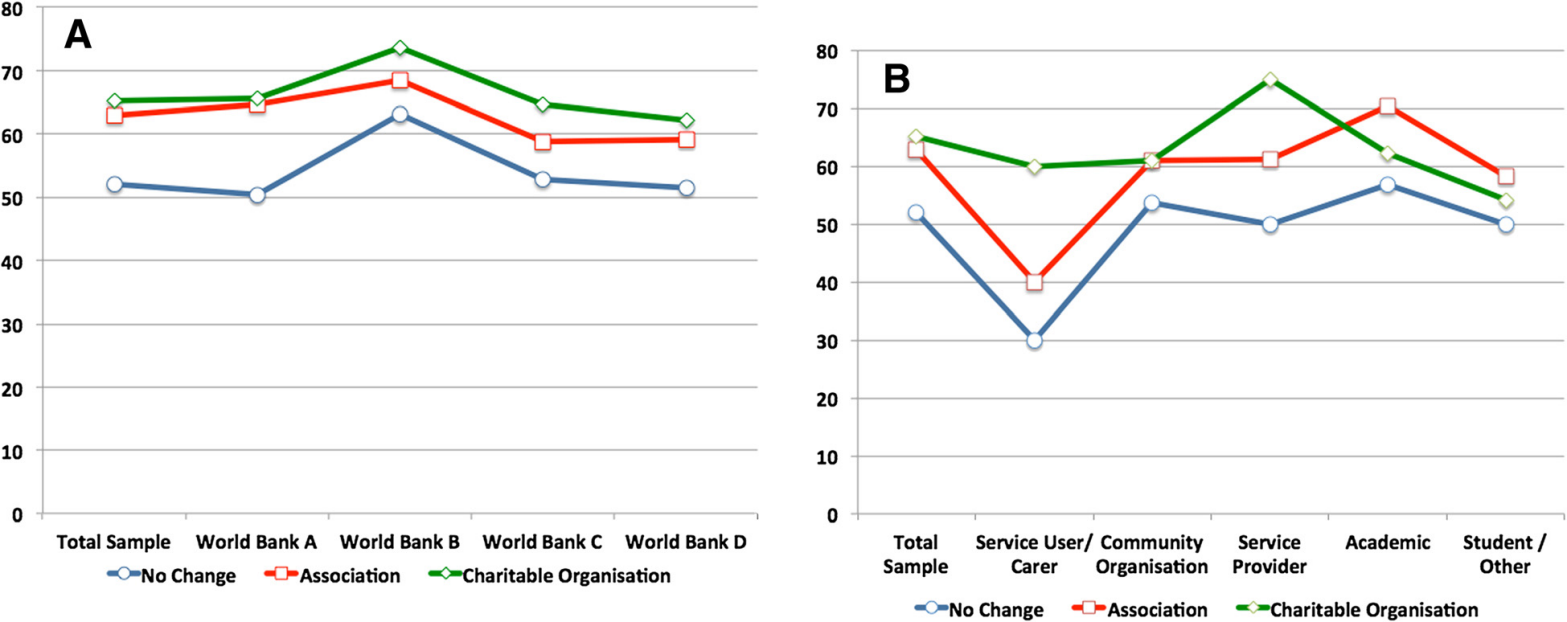
Suitability of each organisational option for pursuit of MGMH goals by World Bank categories of respondents’ countries (Panel A) and respondents’ Primary Affiliations (Panel B).

More than half of the respondents regarded each of the three options as being a suitable form of organisational structure for the pursuit of MGMH goals (Figure 2). The smallest proportion (52%) regarded “No Change” as suitable, the largest proportion (65.2%) regarded “Charitable Organisation” as suitable, with “Association” in between (62.9%). Although there was no significant difference across Affiliation or World Bank groups “No Change” was consistently regarded across all World Bank country categories (Figure 2, Panel A) and Primary Affiliation groups (Figure 2, Panel B) as the least suitable organisational option.

### Ranking of organisational options

**Figure 3:**
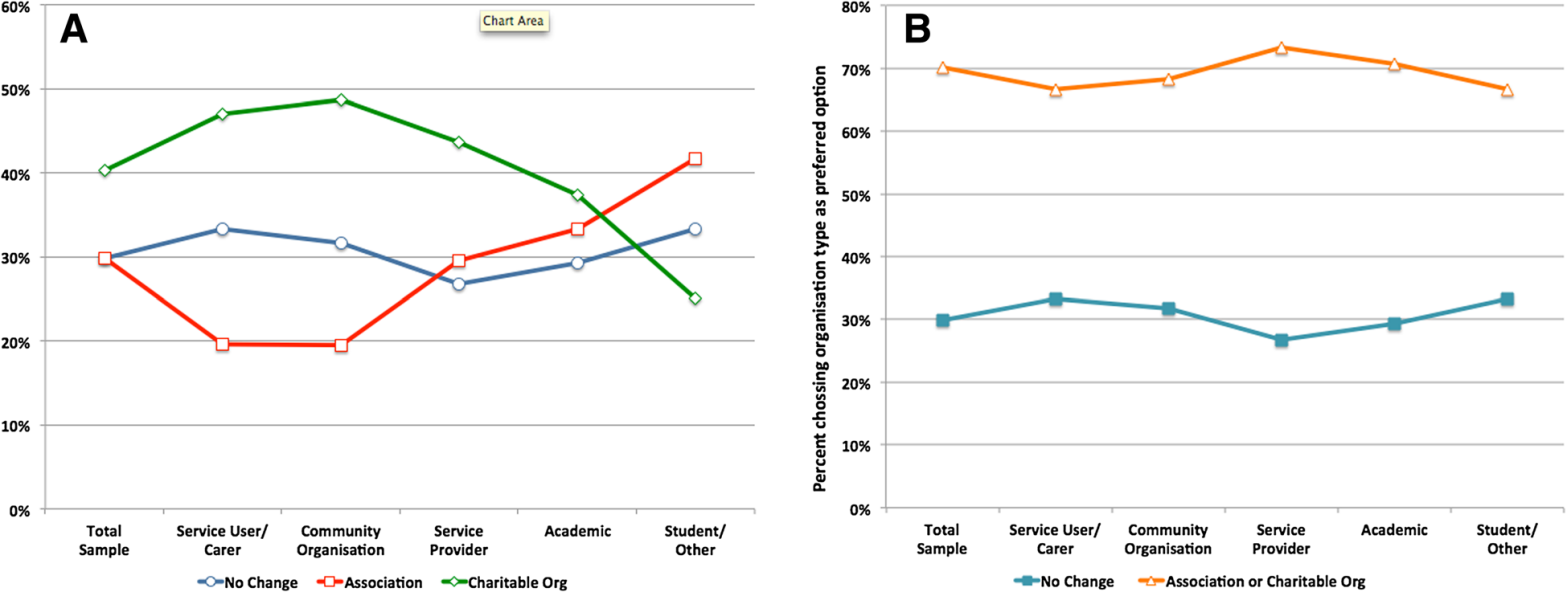
**Percentage of respondents choosing each of the three organisational options as their first preference by Primary Affiliation (Panel A) and percentage of respondents choosing *No Change *** **and formal legal structure (*Association *** **or *Charitable Organisation*) as their first preference by Affiliation (Panel B).**

When ranking the three organizational options in their order of preference, the same number of respondents (66, 29.9%) selected *No Change* and *Association* as their first preferred option for the future organizational structure of MGMH, while 89 (42.2%) selected Charitable Organisation as their first preferred option (Table 2). Less than a third of respondents preferred that the future organizational structure of MGMH should remain unchanged – a loose grouping of interested individuals and institutions around a public website. More than two thirds (70.1%) preferred that a formal legal organisational structure be developed, with 60% of this group preferring a Charitable Organisation and 40% preferring an Association. There were no statistically significant differences in the rankings across the World Bank country categories and primary affiliation groups (Figure 3, Panel A and B).

## Discussion

The number of responses to this survey was small, 7% of individuals registered through the MGMH website. Further, the composition of the sample is skewed. Most respondents were professional service providers or academics from high-income countries. It was not possible to determine how representative of the membership the sample was – in terms of country and primary affiliation - because the relevant information is not collected on registration.

The results of the survey must therefore be treated as a preliminary indication of the views of the MGMH membership. Any conclusions drawn must be provisional, and await clarification from a substantially larger and more representative sample of the MGMH membership. However, the provisional findings raise important questions for the future operation of MGMH. Further investigation of the issues raised should be a high priority for the new Secretariat. There is clearly a need to fully engage service users and carers and MGMH members from low- and middle-income countries in the activities and decision-making processes of the organisation. This will require the new Secretariat to develop strategies for more effective engagement of the whole of the membership and to develop more transparent mechanisms for decision-making. It will also require more complete information about MGMH members to be collected, including information on primary affiliation and country. This will make it possible to track levels of involvement by MGMH members from low- and middle-income countries and members who are service users or carers.

The goals of MGMH have very strong support among the respondents to this survey. However, these goals are broad and there is a need to translate them into specific objectives that are achievable in widely varying political, economic and socio-cultural contexts, with definable indicators of success and clear timelines. There is also a need to focus on objectives that are considered by the organisation as having the highest priority. There are several statements that can be used by MGMH in order to establish priorities. The clearest of these, in terms of relevance for MGMH, is the organisation’s own position statement on mental health in the post-2015 development era [12]. In the area of mental system development the WHO Action Plan [14] has been adopted by the World Health Assembly as a guide to countries on areas of priority development. In the area of research the grand challenges in global mental health [15] is an excellent starting point. However, not everything that needs to be done in pursuit of the broad goals of MGMH can be done by MGMH. This is particularly so if MGMH remains a loose informal organisation without the capacity to generate and manage funds or the capacity to employ staff.

While more than half of the respondents considered each of the three organisational options to be suitable organisational arrangements to pursue the goals of MGMH, *No Change* was the least preferred option for the future organisational structure of MGMH. There was a clear preference for a formal organisational structure, with *Charitable Organisation* preferred over *Association*. If this is the course that is taken it would result in a change in the character of MGMH, which was initially conceived, and has promoted itself, as a social movement [16-18].

Governance arrangements in the current MGMH structure need to be clarified and to be more transparent. Although there is an Advisory Group there is no clarity about who has authority to make decisions on behalf of MGMH. The membership of the Advisory Group has remained virtually unchanged since the establishment of MGMH in 2008. As already mentioned above, the members of the Advisory Group are predominantly the members of the Lancet Global Mental Health Group that produced the first Lancet series in 2007, with a number of additional members joining the group subsequently. Not surprisingly, there is an over-representation in this group of mental health professionals and academics. There is no formal mechanism for appointing or electing members to the MGMH Advisory Group. Moving to a charitable organisation or association structure would require the details of the governance arrangements to be decided and for these arrangements to be fully transparent.

The contributions that MGMH can make in the context of the post-2015 development framework are numerous and exciting. Many of these opportunities, however, remain out of reach while MGMH remains in its current form, which is primarily as an unfunded, international network based around an openly-accessible website. The contributions of individuals and institutions affiliated with MGMH should not be conflated with the contributions that MGMH makes as an organisation. Given that all of the Secretariat work is carried out on a volunteer basis, and the absence of a legal structure that would enable MGMH to receive financial support, employ staff, or establish formal partnerships, the resources needed to act on the above opportunities are lacking.

This lack of resources is not unique to MGMH, and remains a major barrier to scaling up mental health services in countries at all income levels [3]. If MGMH is to help rectify this situation, it will first need to decide how to organise its own resources, whether financial or otherwise, and whether this will require a change to its organisation in the future.

## Conclusions

The global mental health field [12,19,20] and MGMH are in a time of transition. The move to the new secretariat is an opportunity for systematic consideration of the organisational structure and governance arrangements that will best serve the goals of MGMH.

While the respondents to this survey have expressed a clear preference for MGMH to become a formal legally constituted organisation, with a Charitable Organisation structure preferred over an Association, the low rate of response to the survey, particularly among members in low- and middle-income countries and non-health professionals, means that these findings are provisional and do not constitute a firm basis for decision-making about the issue of structure and organisation of MGMH. It will be necessary to go back to the membership and elicit a much more representative response before there can be confidence that decisions concerning structure and organisation represent the considered preferences of the membership.

We do not in this article promote a preferred form of organisational structure for MGMH. Our aim in presenting the results of this survey is to promote a discussion about which organisational and governance arrangements will best serve the objectives of the organisation.

## Competing interests

The authors were members of the MGMH Secretariat when the survey was conceived, carried out and analysed. They wish to particularly state that the views expressed in the article are the views of the authors and should not be interpreted as representing the views of the Movement for Global Mental Health.

## Authors’ contributions

HM conceived the study, AW and HM wrote the first draft and HM wrote the final draft of the manuscript. HM, AW and RK all contributed to the design of the survey, analysis of the data and to drafts of the manuscript. All authors read and approved the final manuscript.
